# Physical Attractiveness and Chances of Being Invited to Interview With a Medical Residency Program: Retrospective Cohort Study

**DOI:** 10.2196/81052

**Published:** 2026-06-04

**Authors:** Daria Hunter, Luke Hunter, David Kerner, Aidan Mullan, Derek Vanmeter, Ivan Khapov, Alexander Finch, Sara Hevesi, Ivan Porter II, Colin West, James Homme

**Affiliations:** 1 Mayo Clinic, 200 1st St SW, Rochester, MN, 55905, United States, 1 (507) 255-5123; 2 Olmsted Medical Center, Rochester, MN, United States; 3 Spartanburg Regional Medical Center, Spartanburg, SC, United States; 4 Geisinger Medical Center, Denvile, PA, United States; 5 Mayo Clinic, Jacksonville, FL, United States

**Keywords:** medical residency, retrospective cohort study, medical residents, attractiveness, residency, interview

## Abstract

**Background:**

Applicants participating in the Residency Match generally submit a photograph through the Electronic Residency Application Service (ERAS). Studies demonstrate that subjectively more attractive applicants are more likely to succeed during job recruitment, including a paper related to the Residency Match.

**Objective:**

This study further investigates the relationship between an applicant’s attractiveness and the likelihood that they are invited to interview with a residency program to explore if more attractive applicants are more likely to be invited to interview when controlled for demographic and academic variables. If there are enough data suggesting that an ERAS photograph being visible prior to the interview gives an unfair advantage to more attractive applicants, this practice might be reconsidered by some residency programs or by ERAS itself.

**Methods:**

Residency directors were surveyed on application review practices. Programs that viewed ERAS photographs prior to deciding whether to invite an applicant to interview were asked to share ERAS files of all reviewed applicants of the 2022 Match. A machine learning model was used to determine attractiveness scores for ERAS photographs. The scores ranged from 1 to 10, where 1 represents the least attractive and 10 represents the most attractive. Multivariable logistic regression analysis was performed considering attractiveness scores, demographics, and professional characteristics. The primary outcome of interest was an invitation to an interview with a residency program.

**Results:**

The residency program response rate was 47.5% (29/61). Among 2681 unique applications to 10 specialties in a single academic health system, the median attractiveness score for all applicants was 6.02 (IQR 5.54-6.55). The univariable analysis indicated a 19% higher invitation likelihood with a 1-point increase in attractiveness. After adjusting for demographics and professional experiences, the association lost statistical significance. Additional adjustment for United States Medical Licensing Examination scores further attenuated the association.

**Conclusions:**

While higher attractiveness scores correlated with an increased likelihood of securing an interview, this correlation was not statistically significant after adjusting for other variables.

## Introduction

The practice of recruitment to medical residencies varies significantly between countries. It ranges from being mostly based on test scores to a series of in-person interviews. However, in most cases, the recruitment of new residents consists of an initial application that may include a professional photograph, followed by an interview in some cases, culminating in a decision on whether an applicant would be a good match. This study focuses on graduate medical education recruitment in the United States due to the location of our institution and US processes being well established and centralized. However, the results of the study may be relevant for other countries and institutions that review applicants’ photographs as part of the residency recruitment process.

Most medical residency positions in the United States are filled via the National Residency Matching Program (NRMP), a system that helps to “match” residency applicants and programs utilizing a proprietary “matching algorithm” that is based on the stable marriage problem—a method of finding an optimal match between 2 equally sized cohorts, given an ordering of preferences for each element of both cohorts [1]. The algorithm takes into account the ranked preferences of both parties and then, through an iterative process, strives to find the match that maximizes overall satisfaction. In 2022, there were 48,156 registered NRMP applicants competing for 37,425 first-year and 2950 second-year positions [2]. Residency applicants create Electronic Residency Application Service (ERAS) applications to apply directly to individual programs. Residency programs use a variety of methodologies to review ERAS applications to select applicants to invite for a formal interview. After interviews are completed, both applicants and programs create Rank Order Lists using the NRMP software. Based on the Rank Order Lists, the matching algorithm pairs applicants and programs. Applicants are notified of the results on Match Day in cases of a successful match. This process is often referred to as “The Match” [2].

Applicants participating in The Match are advised to submit a professional photograph through their online application, along with United States Medical Licensing Examination (USMLE) scores, medical school information, Medical Student Performance Evaluation, letters of recommendation, and curriculum vitae. Public data on what percentage of applicants choose to submit a photograph in their applications are unknown. Based on the data available from our institution, most applicants include a photograph in their application. Programs may voluntarily blind themselves to these photographs through any part of application review, though there are currently no available national data on the utilization of this practice.

Physical attractiveness is generally known as a person’s physical features that are considered esthetically pleasing or beautiful. Robust literature describes the relationship between candidate attractiveness and competitive advantage in a variety of domains, with examples including improved self-image and psychological well-being [3], better treatment by other people [4], higher income [5,6], and even differences in judgment and recommended penalty assessment in a simulated court of law [7]. Studies demonstrate the positive effect of attractiveness on academic performance for trainees [8] and, similarly, on instructor performance evaluations [9,10]. The job market is no exception, as there is evidence that more attractive applicants may be more likely to receive an interview offer [11,12]. Job candidate appearance, including physical attractiveness, attire, posture, and nonverbal communication, plays a significant role in shaping first impressions and can set a competitive advantage affecting hiring outcomes. Available data suggest that physical attractiveness may influence the employment process across multiple fields [11,13-16]. There is substantial empirical evidence that physical attractiveness increases the likelihood of being hired [11,16,17] and might positively affect perception of a candidate’s achievements [18]. This bias, known in psychology as the “beautiful is good” phenomenon or the “halo effect,” has been demonstrated in numerous studies and reviews and found in a variety of different cultures [19-22]. Some evidence suggests that for certain positions traditionally dominated by either male or female candidates, attractive applicants might be evaluated less favorably [23,24].

Hiring in the medical field might differ from hiring in most other professional domains due to the high-stakes nature of clinical work, strict regulatory requirements, and the ethical obligation to prioritize patient safety and competence. NRMP and ERAS platforms were designed to emphasize more objective criteria, including academic and examination performance, evaluations, and extracurricular activities, thereby reducing the influence of superficial characteristics such as physical appearance. Having said that, program directors are often required to review thousands of applications, many of which reflect similar academic metrics, test scores, and clinical experiences. In this context of applicant homogeneity and cognitive overload, subjective factors may regain influence. While the literature on physical attractiveness and its effect on job and academic prospects is abundant, limited data are available in the setting of medical residency. This is despite the fact that the medical residency selection process, unlike many other fields, almost universally utilizes professional photographs as a part of initial applications.

A study published in 2019 by Maxfield et al [25] explored this question in relation to the residency match process. The authors created mock radiology residency match applications utilizing open-access professional photographs. Academic characteristics were randomly assigned to the mock applicants. The authors found that more attractive applicants were likely to receive a higher score from volunteer core faculty members across multiple radiology residency programs than less attractive applicants when controlling for other variables [25]. To our knowledge, this is the only paper investigating the question of physical appearance in medical education and recruitment. There have been no studies of this question utilizing native applications.

While having a professional photograph as part of a residency application serves a clear purpose of making the applications and interviews easier to remember, as residency recruitment committees may go through thousands of applications and interview hundreds of applicants during a single Match season, it is unclear if having a photograph available for review prior to deciding whether to invite an applicant to interview may introduce a bias, as suggested by the results of the publication by Maxfield et al [25]. The goal of this study is to further evaluate the issue of applicants’ physical attractiveness in the residency match interview selection process utilizing native Match data as opposed to mock applications.

## Methods

### Ethical Considerations

This study was approved by the Mayo Clinic Educational Research Committee and the Mayo Clinic Institutional Review Board (IRB), IRB ID 23‐002393. For responding to residency recruitment committee members, a waiver of informed consent was utilized with IRB approval, as only those willing to participate filled out the survey and provided requested data. All participants were informed that they were free to withdraw from the study at any time. For residency applicants, informed consent was obtained by ERAS at the time of registration. Data for both program leadership members and ERAS applicants were anonymized for privacy purposes. Our study complied with ERAS data policy, including only conducting research after the respective Match Day. For the manuscript, we utilized the recommendations for reporting machine learning (ML) analyses in clinical research guidelines [26]. There was no compensation for participating in the study.

### Study Question and Intended Use of the Results

Our goal was to correlate candidate attractiveness as ranked by an ML algorithm, with the likelihood of receiving an interview invitation using primary residency applications (rather than simulated applications). To achieve this goal, we first surveyed 61 program directors from all residency programs in our institution, comprising 3 main academic sites in Minnesota, Arizona, and Florida, and multiple smaller community sites across these states. Twenty-nine program directors volunteered to participate in the survey. Questions in the survey were related to the residency interview selection process and included questions clarifying if the program was blinded to candidate photographs and if candidates’ physical attractiveness was considered when extending interview invitations. As the focus of our research was to evaluate the relationship between physical appearance and chances to be invited to interview, programs that reported waiving their right to view ERAS photographs were not included in the study. Twenty programs that stated that they were not blinded to the photographs were asked to submit ERAS applications, including the photographs of all applicants who were reviewed for interview consideration. Of note, during the study period, the electronic application system used by emergency medicine was the ERAS. During the 2025 to 2026 residency recruitment cycle, this changed to residency centralized application service. Specifically, we only solicited ERAS applications of those applicants who passed initial “filters” and were reviewed holistically. For the study respondents who were surveyed and asked to submit program data, informed consent was implied by their participation in the survey and sharing of ERAS applications. Program leadership teams surveyed were provided with a summary of the study design, including information on the risks and benefits of participating in the research and the right to withdraw from participation at any time. While we provided a general description of the study and research questions, we did not emphasize the main research question. This was done to prevent potential self-selection bias.

For included participants, informed consent for research uses of application materials was obtained by ERAS and NRMP at the beginning of the Match season, which 100% of the applicants who participated in the Match through ERAS signed. We also contacted ERAS directly and confirmed that our specific study was compliant with their data policy. The intended use of the results was to raise the question of whether professional photographs should be available for programs to see prior to the decision of whether to invite a candidate to interview being made.

### Available Data

Ten of the 20 programs provided complete data. From the 10 programs that provided complete data, we received 2694 unique ERAS applications across diverse programs, including diagnostic radiology, emergency medicine, family medicine, obstetrics and gynecology, pediatrics, physical medicine and rehabilitation, radiation oncology, and vascular surgery. There was 1 program in Arizona and 9 at the main academic site in Rochester, MN. From this pool, 13 total applications (<0.005%) were excluded (8 due to the absence of an uploaded photograph and 5 for having uploaded extremely low-resolution images [<50 kB] that prevented any meaningful human and/or computer assessment of attractiveness). The variables of interest, including photographs, USMLE scores, medical school, and demographic data (race, gender, age, visa status), were extracted from the ERAS applications.

### ML Method and Rationale

Since attractiveness is a highly subjective measure, there is no defined gold standard. In addition, individuals of varying demographics and backgrounds may value different features in terms of attractiveness. Therefore, instead of using a relatively small focus group of individuals to manually rank applicants’ attractiveness, we employed an ML algorithm that was trained on ratings by hundreds of individuals with distinct backgrounds, incorporating the opinions of a much larger and more diverse group of human raters.

We utilized the MEBeauty Python software package to create a race-sensitive ML model (meaning that the model determines the race of the person in each image and compares it within its race rather than mixing it with all races, given different facial structures and beauty standards between racial groups) that could autonomously estimate attractiveness scores for human faces in photographs. The software package includes a database of 2550 images of Black, Asian, Caucasian, Hispanic, Indian, Mideastern, male, and female faces, scored from 1 (least beautiful) to 10 (most beautiful) by approximately 300 individuals with diverse cultural and social backgrounds. According to the original paper, the scores for each image assigned by different raters fell between 1.5 and 2 SDs [27].

### Evaluation Measures, Training Protocols, and Validation

Using default MEBeauty parameters, 2681 images were preprocessed by a multitask cascaded convolutional neural network that standardized the images by vertically aligning and cropping faces from the full image.

**Figure 1.**
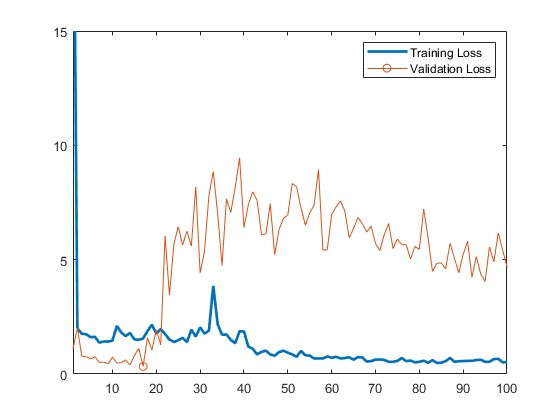
Training and validation loss for each epoch using the VGG-16 model.

Model training and validation were performed to identify the best prediction model and to avoid overtraining or undertraining. Native MEBeauty code was used to perform this task. All models processed 16 images at a time and iteratively refined the prediction of attractiveness using a maximum of 100 passes on the training data. The use of each database image as either a training or a validation image was predetermined by the MEBeauty database. Therefore, the model was developed using an existing database of images and then applied to the applicants’ photographs. Several different model types were assessed: VGG-16, MobileNet, DenseNet, and AlexNet. All models are open-access convolutional neural networks that are commonly used for image classification, but not exclusively for facial images. Therefore, several different models were trained and tested to select the best-performing network for the purposes of this paper. There was broad ranking agreement between the different candidate model types, with the VGG-16 yielding the best performance on validation images (validation loss 0.329, Figure 1) when compared with the other model types (MobileNet=0.940, DenseNet=0.510, and AlexNet=0.581). Therefore, this VGG-16 model was chosen as the facial beauty score predictor for the novel residency applicant images assessed that we acquired and assessed. Physical attractiveness scores were assigned to each applicant based on model prediction, and these data were combined with the rest of the variables extracted from the ERAS applications.

### Measurements and Outcomes

The primary outcome of interest was whether a residency applicant was invited to an interview with a program. Applicant photographs were obtained from the ERAS applications, and attractiveness was determined using the trained ML model. Applicant characteristics, including age, gender, race, residency region (Minnesota, Midwest United States, and non-Midwest United States), visa status, USMLE scores, and relevant research or volunteer experience, were also retrieved from ERAS applications.

All applicants were divided into 2 cohorts: those who were invited to interview with one of the residency programs participating in this research and those who were not. We did not consider whether an applicant accepted the interview offer. In our sample, less than 1% of applicants interviewed with more than one participating residency program.

### Statistical Analysis

Numeric features were summarized with medians and IQRs; categorical features were summarized with frequency counts and percentages. For USMLE score analysis, we used national averages as a cutoff. Exploratory analysis compared applicant physical attractiveness based on applicant education, research or volunteer experience, visa status, and USMLE scores using 2-sided Kruskal-Wallis tests.

The association between artificial intelligence (AI)–determined physical attractiveness and invitation to interview was assessed using logistic regression. Models were unadjusted, adjusted only for the applicants’ demographics (age, gender, race, and address location categorized as Minnesota, Midwest US, and non-Midwest US), and fully adjusted for demographics, medical credentials (Medical Doctor [MD] degree, Doctor of Osteopathic Medicine [DO] degree, or International Medical Graduate), relevant experience (number of research opportunities, number of volunteer opportunities), and USMLE step 1 and step 2 test scores. Model results were reported using odds ratios (ORs) with 95% CIs. The association between invitation to interview and all applicant characteristics included in the fully adjusted model was reported in addition to the association with applicant attractiveness.

A minority of applicants did not report USMLE step 1 (16.9%) or USMLE step 2 (11.1%) or both scores and were excluded from this model. Sensitivity analysis included the applicants with missing USMLE scores as a standalone “Not Reported” group.

Secondary analysis considered each residency specialty individually when assessing the impact of AI-attractiveness on being invited to interview. Only candidates reviewed for each specialty were included. All specialties were considered during univariable analysis. To ensure that the adjusted regression models had sufficient statistical power, only specialties with at least 300 reviewed candidates were included for multivariable analysis.

Statistical significance was set at a threshold of *α*=.05. All analyses were conducted using R (version 4.2.2; R Foundation for Statistical Computing).

## Results

### General Results

Twenty-nine program directors out of 61 (47.5%) volunteered to participate in the survey. Of the 29 respondents, 20 indicated that their recruitment committee was not blinded to the applicants’ photographs. All 29 respondents indicated that they did not consider applicants’ physical appearance when deciding whether to invite them to interview. Ten programs provided complete applicant data, which were included in the final analysis.

A total of 2681 candidates were reviewed for at least one residency program. Across all specialties that participated in the study, the median applicant age was 27 (IQR 26‐29) years, 1351 (50.4%) applicants were women, 1346 (50.2%) self-identified as White, and 184 (6.9%) required visa sponsorship. The median step 2 score was 249 (IQR 239‐259), which is consistent with the national average [28]. For other academic characteristics, study applicants were similar to the national averages as reported by the NRMP [29-31] and are summarized in Table 1.

**Table 1.** Summary of applicant demographics, United States Medical Licensing Examination scores, and experiences (N=2681).

Demographics	Values
Age (y), median (IQR)	27 (26-29)
Gender, n (%)	
Women	1351 (50.4)
Men	1322 (49.3)
Other	7 (0.3)
Unknown	1 (0.0)
Race, n (%)	
American Indian	31 (1.2)
Asian	462 (17.2)
Asian Indian	283 (10.6)
Black or African American	169 (6.3)
Hispanic	208 (7.8)
White	1346 (50.2)
Other	182 (6.8)
Applicant type, n (%)	
MD^a^	1950 (72.7)
DO^b^	407 (15.2)
Non-US IMG^c^	237 (8.8)
US IMG	85 (3.2)
Other	2 (0.1)
Address location, n (%)	
Minnesota	143 (5.3)
Midwest (MN^d^, IA^e^, WI^f^, ND^g^, SD^h^)	343 (12.8)
Home institution affiliation, n (%)	
Main institutional location	15 (0.6)
Across all sites of the institution	26 (1.0)
Authorized to work in the United States, n (%)	
No	106 (4.0)
Yes	2575 (96.0)
Visa sponsorship needed, n (%)	
No	24 (0.9)
Yes	184 (6.9)
Unknown/not applicable	2473 (92.2)
AI^i^-determined physical attractiveness**,** 1‐10, median (IQR)	6.02 (5.54-6.55)
USMLE^j^ scores and relevant experience	
Step 1 attempted, n (%)	
No	274 (10.2)
Yes	2407 (89.8)
Step 1 score, median (IQR)	238 (224-248)
Step 2 CK^k^ attempted, n (%)	
No	299 (11.2)
Yes	2382 (88.8)
Step 2 CK score, median (IQR)	249 (239-259)
Step 2 CS^l^ attempted, n (%)	
No	2589 (96.6)
Yes	92 (3.4)
Step 2 CS result, n (%)	
Pass	92 (100)
Fail	0 (0)
Step 3 attempted, n (%)	
No	2555 (95.3)
Yes	126 (4.7)
Step 3 score, median (IQR)	226 (212-236)
Number of research experiences, median (IQR)	3 (2-5)
Number of volunteer experiences, median (IQR)	8 (5-11)

a MD: Doctor of Medicine.

b DO: Doctor of Osteopathic Medicine.

c IMG: International Medical Graduate.

d MN: Minnesota.

e IA: Iowa.

f WI: Wisconsin.

g ND: North Dakota.

h SD: South Dakota.

i AI: artificial intelligence.

j USMLE: United States Medical Licensing Examination.

k CK: clinical knowledge.

l CS: clinical skills.

**Figure 2.**
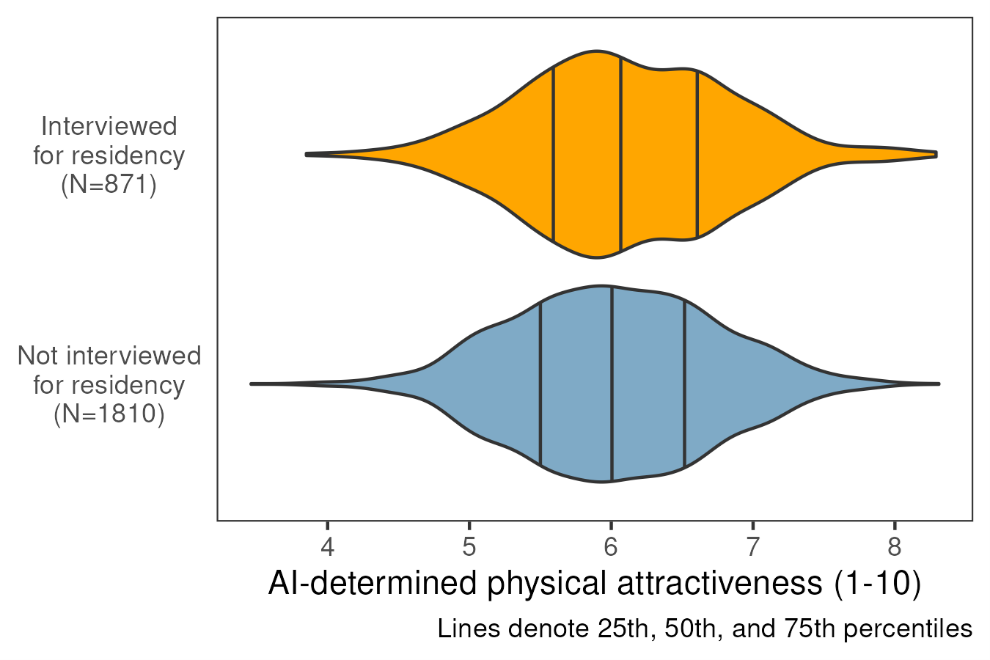
Artificial intelligence (AI)–determined physical attractiveness among candidates interviewed and not interviewed for medical residency.

Of the 2681 reviewed applications, 871 candidates were invited to an interview. The median AI-determined attractiveness score among all candidates was 6.02 (IQR 5.54‐6.55). AI-determined physical attractiveness score distribution among candidates interviewed and not interviewed for medical residency is available in Figure 2.

### ML Attractiveness Grading

MEBeauty AI is subject to the same human biases as the people who trained it, as described in the original paper [27]. In our data, male candidates were assigned lower attractiveness scores than female candidates, with the median score for men being 5.67 (IQR 5.27-6.02) and for women being 6.48 (IQR 6.0-6.84), *P*<.001. In addition, White applicants received slightly higher scores than non-White applicants (median score for White candidates was 6.07, IQR 5.61-6.58, and for non-White candidates was 5.98, IQR 5.48-6.50; *P*<.001), although the difference was very small.

### Association Between Attractiveness and Interview Invitation

Univariable logistic regression found that a 1-point increase in attractiveness was associated with 19% higher odds of being invited to interview for residency (OR 1.19, 95% CI 1.06‐1.33; *P*=.003). However, when only adjusting for applicant demographics (age, gender, race, and address), there was no significant association between attractiveness and being selected to interview (OR 1.06, 95% CI 0.92‐1.22; *P*=.45). Further adjusting for applicant certification, research and volunteer experience, and USMLE scores similarly found no association between attractiveness score and interview invitation (OR 1.01, 95% CI 0.86‐1.20; *P*=.87; Table 2).

**Table 2.** Multivariable associations between applicant characteristics and being invited to interview for any specialty.

Applicant characteristic	Odds ratio (95% CI)	*P* value
Age, per year	1.06 (1.02-1.10)	.003
Gender (reference: women)		
Men	0.73 (0.57-0.95)	.02
Race (reference: White)		
Not White	1.02 (0.83-1.26)	.86
Applicant credentials (reference: no MD^a^)		
MD	2.74 (2.03-3.70)	<.001
Address (reference: Minnesota)		
In the Midwest, non-Minnesota	0.31 (0.17-0.56)	<.001
Outside the Midwest	0.13 (0.08-0.20)	<.001
Authorized to work	1.22 (0.66-2.25)	.52
Research experience, per experience	1.05 (1.01-1.10)	.02
Volunteer experience, per experience	1.05 (1.02-1.07)	<.001
USMLE^b^ step 1 score, per 10 points	1.04 (0.95-1.14)	.42
USMLE step 2 CK^c^ score, per 10 points	1.30 (1.17-1.45)	<.001
AI^d^ attractiveness, per 1 point	1.01 (0.86-1.20)	.87

a MD: Doctor of Medicine.

b USMLE: United States Medical Licensing Examination.

c CK: clinical knowledge.

d AI: artificial intelligence.

A sensitivity analysis including applicants with missing USMLE scores as a separate “not reported” group found similar results (OR 1.05, 95% CI 0.91‐1.23; *P*=.49). When only adjusting for applicant academic and visa credentials other than USMLE scores (being an MD as opposed to DO, authorized to work in the United States, number of research experiences, and number of volunteer experiences), there was similarly no statistically significant association between attractiveness and getting an interview (OR 1.10, 95% CI 0.98‐1.25; *P*=.11).

### Association Between Applicant Characteristics and Interview Invitation

On the multivariable analysis including USMLE scores, we found that male candidates were less likely to be invited to interview than female candidates (OR 0.73, 95% CI 0.57‐0.95; *P*=.02) and MD candidates were more likely to be interviewed than DOs and International Medical Graduates (OR 2.74, 95% CI 2.03‐3.70; *P*<.001). The vast majority of analyzed ERAS applications came from Minnesota (9/10 residency programs), and applicants from the Midwest excluding Minnesota (where our institution is located) were less likely to be selected to interview (OR 0.31, 95% CI 0.17‐0.56; *P*<.001) compared to candidates from Minnesota, as were applicants outside of the Midwest (OR 0.13, 95% CI 0.08‐0.20; *P*<.001). There was a 6% increase in the odds of receiving an interview invitation per additional year of age (OR 1.06, 95% CI 1.02‐1.10; *P*=.003), a 5% increase per additional research experience (OR 1.05, 95% CI 1.01‐1.10; *P*=.02), a 5% increase per additional volunteer experience (OR 1.05, 95% CI 1.02‐1.07; *P*<.001), and a 30% increase per 10 additional points on the USMLE step 2 clinical knowledge (CK) score (OR 1.30, 95% CI 1.17‐1.45; *P*<.001). Race, work authorization in the United States, and USMLE step 1 score did not demonstrate statistically significant associations with being selected to interview.

### Association Between Attractiveness and Other Variables

Examining attractiveness across applicant credentials, we found that MD candidates were rated as more attractive (median attractiveness 6.06 vs 5.94; Kruskal-Wallis *P*<.001), candidates with more research were more attractive (0‐3 vs 4+ research experiences: median attractiveness 6.08 vs 5.98; *P*<.001), and candidates with more volunteering were more attractive (9+ vs 0‐8 volunteer experiences: median attractiveness 6.19 vs 5.91; *P*<.001). There was no correlation between attractiveness and being authorized to work in the United States (median attractiveness 6.02 vs 6.01; *P*=.61). There was a small negative association between AI-attractiveness and USMLE step 1 score (step 1 score 239+ vs <239; median attractiveness 5.95 vs 6.10; *P*<.001), but there was no association between AI-attractiveness and USMLE step 2 CK score (step 2 CK score 250+ vs <250; median attractiveness 6.05 vs 6.00; *P*=.10) or step 3 score (step 3 score 227+ vs <227; median attractiveness 5.95 vs 5.79; *P*=.52).

When assessing program-specific data, some programs were demonstrated to be more likely to invite more attractive applicants to interview, but after adjusting for demographic and objective academic parameters, no association of physical attractiveness with invitation to interview was found across programs.

## Discussion

### General Discussion

This study of native ERAS applications across 10 residency programs in an academic institution with locations in Minnesota and Arizona demonstrates an association between physical appearance and chances of being invited to interview with a residency program that disappears when adjusted for demographic and academic characteristics. While our research demonstrated a statistically significant unadjusted correlation between physical attractiveness and chances of being invited to a medical residency interview, this association was small and disappeared after adjusting for academic and demographic variables.

Our work builds on the contributions of others in this field. Maxfield et al [25] randomly assigned academic characteristics to open-access professional photographs and demonstrated a bias in favor of more attractive applicants when controlled for objective variables such as USMLE step scores. Regarding demographic variables, Maxfield et al [25] considered race or ethnicity and gender but not the age of the applicants. They demonstrated that reviewers preferred Black and Hispanic applicants relative to White or Asian applicants. There were no significant interactions between race or gender and attractiveness. Although a different methodology was utilized, our data demonstrate a relatively small difference in attractiveness between applicants invited and not invited to interview, while in the study by Maxfield et al [25], physical appearance was the second strongest predictor of being invited to interview after the USMLE step 1 score. This could be related to the fact that Maxfield et al [25] utilized stock images of mock applicants while we used native applications, or it could be attributed to the AI’s tendency to regress to the mean. Of note, another study by Steiner et al [32] demonstrated opposite findings in terms of race-related outcomes, finding that White program directors were more likely to match White applicants.

We believe that our data extend this prior work. While our research shows an apparent initial association between physical appearance and the chances of being invited to interview with a residency program, it demonstrates that this association disappears when adjusted for demographic and academic characteristics. This result indicates that attractiveness is likely a confounder linked with other factors such as demographics and gender relating to interview selection rather than a main variable independently contributing to interview selection.

The findings that MD candidates and candidates with more research and volunteer experiences are assigned higher attractiveness scores also suggest that, while physical appearance may not be a major factor in residency interview selection, it may provide an advantage through applicants’ career development. Interestingly, USMLE scores either did not correlate or negatively correlated with attractiveness, which is thought-provoking as, unlike getting accepted to an MD-granting medical school and securing volunteer or research experiences, USMLE performance does not require interaction with another human via interview or collaboration. These results further point to a potential advantage that more attractive people have when it comes to academic achievements that rely on interpersonal communication rather than just medical knowledge alone. It is also aligned with other studies focused on the mostly positive impact of physical attractiveness on academic and career success [5,6,8,10-12,17,18].

The fact that we observed no difference in interview offers when controlled for demographic and academic characteristics suggests that the ERAS application process is unlikely to directly contribute to biased interview selection if a professional photograph is available to residency selection committees prior to deciding to invite an applicant to interview. Similarly, for residency programs outside of the United States, based on our data, use of professional photographs in the initial application is unlikely to give an unfair advantage to more attractive applicants. Put another way, our results do not support a causative link between photographic attractiveness and propensity to receive an interview invite. However, our data, in the context of the multidisciplinary work referenced previously, continue to raise questions as to the benefits of increased attractiveness compounded over a career, which may yield multifactorial benefits resulting in improved academic and career prospects.

To our knowledge, ours is the first work that combines ML methodology in combination with native applications in a real-life application cycle to explore this important topic. One study has explored algorithms incorporating traditional residency application metrics and applicant demographics, demonstrating high correlation with match success, although that study did not focus on the photograph [33]. Traditionally, studies focusing on the effect of physical appearance have utilized a group of individuals to grade attractiveness. We suggest that utilization of an AI algorithm may be a more objective way to achieve this goal. Since AI tools are often trained on hundreds or thousands of people, they may be less prone to individual biases. Nonetheless, because AI machines are trained to replicate real people’s opinions, there have been multiple reports demonstrating biases in AI’s grading of attractiveness, such as preferring Caucasian race and female sex [34,35]. This can be minimized, although not eliminated, by using culturally diverse AIs such as MEBeauty that we selected for this project. We reviewed multiple AI algorithms and training datasets and chose MEBeauty due to its diverse range of ethnicities, ages, genders, and unrestricted facial expressions. In addition, not only did the training dataset include thousands of pictures of diverse people, but hundreds of individuals who graded the images to train the AI were also specifically selected to represent various gender, ethnic, and age backgrounds [27].

### Limitations

This is a single-center study with a limited number of residency program participants. While the number of ERAS applications is more than 2000, it constitutes only a small percentage of the total number of annual residency match participants. In addition, all programs were from only 2 geographic locations, which may represent local practice and not be representative of other programs in the United States. Our sample objective data, such as gender, USMLE scores, number of volunteer experiences, and number of research experiences, are nearly identical to national averages. This suggests, but cannot guarantee, representativeness in utilizing our data to provide some prediction of the impact of physical attractiveness on interview invitation within the larger applicant pool for the 2022 residency match. In addition, participating programs self-reported on blinding to ERAS photographs and the role of physical attractiveness in their interview selection process. These reported data may not represent actual practice; however, all programs were provided with a general description of the project without specific details on the main research hypothesis and were reported in aggregate to encourage accurate reporting. We intentionally did not report program-specific data to preserve program and applicant confidentiality.

Unfortunately, some of the original raw data are no longer available. This includes code that was used to train and validate the model, the specific loss function used, correlation metrics between predicted and ground-truth scores on the validation set, mean absolute error in interpretable units, the specific version of MEBeauty/PyTorch code used, the random seed for reproducibility, the preprocessing parameters applied to ERAS photographs, and whether the pretrained VGG-16 weights were ImageNet-initialized or trained from scratch.

We used professional photograph image files as the main metrics for quality of the photograph. While ERAS photographs are highly standardized in terms of applicant position and attire, there are some differences in facial expression, eyewear use, background color, and hairstyle that we were not able to assess given the large amount of data. No validation was performed on a subset of ERAS photos to confirm that the model generalizes appropriately to this new domain.

We utilized race-sensitive AI to reduce bias, as program committee members who responded to the survey were representatives of a variety of racial backgrounds. However, we did not specifically tailor the AI to assess applicants based on a specific program director’s race. This was due to the fact that the selection process often involves multiple committee members, such as program directors, associate program directors, and program coordinators, and there is no reliable method to distinguish which applicant was invited according to the input of any individual faculty member. Finally, while we used race-sensitive AI, ML models are notorious for being biased, and it is possible that the algorithm we utilized introduced its own bias to this study.

### Conclusion

In our study of a diverse sample of applicants to multiple programs within a large graduate medical education institution, there was no association between a higher AI-generated physical attractiveness score of an applicant and the likelihood of receiving an invitation to interview when controlled for other variables. This suggests that more attractive applicants do not have an advantage in the residency interview selection process simply due to their ERAS photograph. There could, however, be an advantage of being overall more successful academically for more attractive applicants, which indirectly leads to better chances of being interviewed for a residency position, because based on the results of this study, more attractive candidates were more likely to be US MDs and have a higher number of research and volunteer experiences, which are traditionally correlated with higher chances of being invited to interview as per NRMP data [2]. Future areas of research should seek to further evaluate the correlation of attractiveness and residency match while attempting to evaluate potential benefits of attractiveness compounded over a career and include multicenter, geographically diverse settings.

## Abbreviations

AI: artificial intelligence
CK: clinical knowledge
DO: Doctor of Osteopathic Medicine
ERAS: Electronic Residency Application Service
IRB: institutional review board
MD: Doctor of Medicine
ML: machine learning
NRMP: National Residency Matching Program
OR: odds ratio
USMLE: United States Medical Licensing Examination

## References

[1] McVitie DG, Wilson LB (1971). The stable marriage problem. Commun ACM.

[2] (2023). Results and data: 2023 main Residency Match®. https://www.nrmp.org/wp-content/uploads/2023/05/2023-Main-Match-Results-and-Data-Book-FINAL.pdf.

[3] Datta Gupta N, Etcoff NL, Jaeger MM (2016). Beauty in mind: the effects of physical attractiveness on psychological well-being and distress. J Happiness Stud.

[4] Snyder M, Tanke ED, Berscheid E (1977). Social perception and interpersonal behavior: on the self-fulfilling nature of social stereotypes. J Pers Soc Psychol.

[5] Scholz JK, Sicinski K (2015). Facial attractiveness and lifetime earnings: evidence from a cohort study. Rev Econ Stat.

[6] Parrett M (2015). Beauty and the feast: examining the effect of beauty on earnings using restaurant tipping data. J Econ Psychol.

[7] Efran MG (1974). The effect of physical appearance on the judgment of guilt, interpersonal attraction, and severity of recommended punishment in a simulated jury task. J Res Pers.

[8] Hansen K (2016). The relationship between teacher perceptions of pupil attractiveness and academic ability. Br Educ Res J.

[9] Westfall R, Millar M, Walsh M (2016). Effects of instructor attractiveness on learning. J Gen Psychol.

[10] Riniolo TC, Johnson KC, Sherman TR, Misso JA (2006). Hot or not: do professors perceived as physically attractive receive higher student evaluations?. J Gen Psychol.

[11] Watkins LM, Johnston L (2000). Screening job applicants: the impact of physical attractiveness and application quality. Int J Sel Assess.

[12] Agthe M, Spörrle M, Maner JK (2011). Does being attractive always help? Positive and negative effects of attractiveness on social decision making. Pers Soc Psychol Bull.

[13] McElroy JC, DeCarlo TE (1999). Physical attractiveness on cognitive evaluations of saleswomen's performance. J Mark Theory Pract.

[14] Wu PPC, Hwang IS (2012). The influence of gender dyads and physical appearance on the strength of the customer-provider relationship in the Taiwanese hairdressing industry. Serv Mark Q.

[15] Jin N, Merkebu J (2015). The role of employee attractiveness and positive emotion in upscale restaurants. Anatolia.

[16] Dipboye RL, Fromkin HL, Wiback K (1975). Relative importance of applicant sex, attractiveness, and scholastic standing in evaluation of job applicant resumes. J Appl Psychol.

[17] Johnson SK, Podratz KE, Dipboye RL, Gibbons E (2010). Physical attractiveness biases in ratings of employment suitability: tracking down the “beauty is beastly” effect. J Soc Psychol.

[18] Chia RC, Allred LJ, Grossnickle WF, Lee GW (1998). Effects of attractiveness and gender on the perception of achievement-related variables. J Soc Psychol.

[19] Dion K, Berscheid E, Walster E (1972). What is beautiful is good. J Pers Soc Psychol.

[20] Langlois JH, Kalakanis L, Rubenstein AJ, Larson A, Hallam M, Smoot M (2000). Maxims or myths of beauty? A meta-analytic and theoretical review. Psychol Bull.

[21] Zebrowitz LA, Franklin RG (2014). The attractiveness halo effect and the babyface stereotype in older and younger adults: similarities, own-age accentuation, and older adult positivity effects. Exp Aging Res.

[22] Dion KK, Rhodes G, Zebrowitz LA (2002). Facial Attractiveness: Evolutionary, Cognitive, and Social Perspectives.

[23] Cash TF, Gillen B, Burns DS (1977). Sexism and beautyism in personnel consultant decision making. J Appl Psychol.

[24] Cash TF, Kilcullen RN (1985). The eye of the beholder: susceptibility to sexism and beautyism in the evaluation of managerial applicants. J Appl Soc Psychol.

[25] Maxfield CM, Thorpe MP, Desser TS (2019). Bias in radiology resident selection: do we discriminate against the obese and unattractive?. Acad Med.

[26] Stevens LM, Mortazavi BJ, Deo RC, Curtis L, Kao DP (2020). Recommendations for reporting machine learning analyses in clinical research. Circ Cardiovasc Qual Outcomes.

[27] Lebedeva I, Guo Y, Ying F (2022). MEBeauty: a multi-ethnic facial beauty dataset in-the-wild. Neural Comput Applic.

[28] (2022). USMLE score interpretation guidelines. https://www.usmle.org/sites/default/files/2022-05/USMLE%20Step%20Examination%20Score%20Interpretation%20Guidelines_5_24_22_0.pdf.

[29] (2022). Charting outcomes in the match: senior students of U.S. MD medical schools: characteristics of U.S. MD seniors who matched to their preferred specialty in the 2022 main Residency Match. https://www.nrmp.org/wp-content/uploads/2026/03/Charting-Outcomes-MD-Seniors-2022_Final_revised-citation.pdf.

[30] (2022). Charting outcomes in the match: senior students of U.S. DO medical schools characteristics of senior students of U.S. DO medical schools who matched to their preferred specialty in the 2022 main Residency Match. https://www.nrmp.org/wp-content/uploads/2022/07/Charting-Outcomes-DO-Seniors-2022_Final.pdf.

[31] (2022). Charting outcomes in the match: International Medical Graduates: characteristics of International Medical Graduates who matched to their preferred specialty in the 2022 main Residency Match. https://www.nrmp.org/wp-content/uploads/2026/03/Charting-Outcomes-IMG-2022_Final_revised-citation.pdf.

[32] Steiner Q, Edalatpour A, Seitz AJ, Bentz ML, Afifi AM (2023). Diversity in the plastic surgery match: the effect of program chair, program director, and faculties' race and sex on matched applicants. J Craniofac Surg.

[33] Shaffrey EC, Moura SP, Wirth PJ (2023). Objective residency applicant assessment using a linear rank model. J Surg Educ.

[34] Makhortykh M, Urman A, Ulloa R (2021). Advances in Bias and Fairness in Information Retrieval.

[35] Fuchs DJ (2018). The dangers of human-like bias in machine-learning algorithms. Missouri S&T’S Peer to Peer.

